# Multi-stakeholder Engagement for the Sustainable Development Goals: Introduction to the Special Issue

**DOI:** 10.1007/s10551-022-05192-0

**Published:** 2022-09-01

**Authors:** G. Abord-Hugon Nonet, T. Gössling, R. Van Tulder, J. M. Bryson

**Affiliations:** 1 Jönköping International Business School, Region Småland, Jönköping, Sweden; 2 Present Address: Centre of excellence for Sustainability, KEDGE Business School, Bordeaux, France; 3 Department of Business-Society Management, Rotterdam School of Management, Rotterdam, The Netherlands; 4 Hubert H. Humphrey School of Public Affairs, University of Minnesota, Minneapolis, USA

**Keywords:** Sustainable Development Goals, Principles-based approach, Multi-stakeholder engagement, Cross-sector partnerships, CSR, Wicked problems, Hybrid governance

## Abstract

The world is not on track to achieve Agenda 2030—the approach chosen in 2015 by all UN member states to engage multiple stakeholders for the common goal of sustainable development. The creation of the 17 Sustainable Development Goals (SDGs) arguably offered a new take on sustainable development by adopting hybrid and principle-based governance approaches, where public, private, not for profit and knowledge-institutions were invited to engage around achieving common medium-term targets. Cross-sector partnerships and multi-stakeholder engagement for sustainability have consequently taken shape. But the call for collaboration has also come with fundamental challenges to meaningful engagement strategies—when private enterprises try to establish elaborate multi-stakeholder configurations. How can the purpose of businesses be mitigated through multi-stakeholder principle-based partnerships to effectively serve the purpose of a common sustainability agenda? In selecting nine scholarly contributions, this special issue aims at advancing this discourse. To stimulate further progress in business studies, this introductory essay, furthermore, identifies three pathways for research on multi-stakeholder engagement processes in support of the Decade of Action along three coupling lines: multi-sector alignment (relational coupling), operational perception alignment (cognitive coupling) and goal and strategic alignment (material coupling).

## The Context: The Growing Importance of Principle-Based Approaches

The adoption of 17 Sustainable Development Goals (SDGs) in September 2015 by all 193 United Nations member countries, created a common framework for sustainability—Agenda 2030. The SDGs were adopted as a universal call to action to end poverty, protect the planet, and ensure that by 2030 all people enjoy peace and prosperity. Almost all countries agreed on “a shared blueprint for peace and prosperity for all people and the planet” (https://sdgs.un.org/goals). The formation and creation of the goals also portrays the most inclusive and participatory global approach to strategy formulation and development to date. The goals were established following a massive, three years’ process of global multi-stakeholder consultation in which hundreds of big and small corporations, governments, civil society groups, knowledge institutes and other organizations participated. The SDGs can, therefore, arguably, be considered the most all-encompassing, ambitious, as well as action-oriented agenda for progress on a global scale, ever agreed upon by humankind. This does not mean, however, that the SDGs are free of flaws or criticism, but they do point to a way forward for addressing humanity’s sustainability challenges in the future (Van Tulder & Van Mil, 2023).

The common aspiration of the SDGs also signaled the increased recognition of the urgency to take a ‘systems approach’ to sustainable development (‘grand’) challenges. These challenges include ending poverty and other deprivations while developing strategies to improve health and education, reduce inequality, support economic development, and addressing the climate crisis urgency, as well as the critical need to protect and regenerate lands, waters and biosphere—almost at the same time. In an increasingly complex world facing major challenges, the adoption of the 17 Goals should help build capacity, strategy, commitment, and engagement to address humanity’s most threatening challenges in an inclusive manner.

The SDGs introduced a ‘principles-based’ and a ‘governing through goals’ (Kanie and Biermann 2017) based approach to sustainable development. The 17 goals and 169 targets selected are guided by five basic principles (People, Planet, Prosperity, Peace and Partnering) and one overarching principle ‘no one left behind’.

To realize the SDGs, the active participation of corporations was considered vital. This recognition also triggered a form of ‘hybrid’ or ‘transition’ governance for development guided by the common good on a global scale that has not been tried before. A ‘hybrid governance’ approach can be designed with representatives of the public, private, not-for-profit sectors and also knowledge institutions working together to achieve goals through targeted action (van Tulder 2021). As such, the hybrid governance structure of the SDG approach was intended to channel progress in several concrete areas by means of goal prioritization, improved narratives to facilitate broad awareness and commitment, better data development, and instilling of active participation in, for instance, joint research or the creation of new platforms and partnerships around the implementation of the common agenda. This set-up signaled a considerable shift away from traditional ways of thinking about sustainable development as the prime responsibility of governments. Companies—whether big and small, local, and multinational, in all sectors of society—thereby have a crucial role to play, according to the UN: “We acknowledge the role of the diverse private sector, ranging from micro-enterprises to cooperatives to multinationals […] in the implementation of the new Agenda” (United Nations, 2015: 10).

Companies took up the challenge for a variety of ethical and strategic reasons. By 2016, 87% of CEOs believed that the SDGs provide an opportunity to rethink approaches to sustainable value creation, while 78% already recognized opportunities to contribute through integrating the SDGs into their core business (UNGC and Accenture Strategy 2016). However, as van der Waal and Thijssens (2020) point out, there seems to be a tendency to subscribe to SDGs rather symbolically and strategically primarily for legitimacy reasons. In a similar vein, Gneiting and Mhlanga (2021) suggest that companies aim at realizing reputational gains from their SDP-related activities rather than significantly contributing to them. Notwithstanding these early critical observations, company representatives replied that the SDGs promised to unlock an estimated annual US$12 trillion in business investment opportunities (Business & Sustainable Development Commission, 2017) for companies able to come up with innovative sustainable solutions and inclusive business models. The alliance between the UN and major companies also reiterated the importance of a distinct dimension that had not been embraced earlier in initiatives around sustainability issues: rather than a ‘rule-based’ approach to sustainability (stressing laws, codes of conduct and treaties), did the SDG approach embraced ‘principles-based’ management practices as agreed upon in the decade before the SDGs materialized. This included frameworks partly initiated by companies themselves or in coalitions with other stakeholders, like the Ten Principles of UN Global Compact, the Guiding Principles on Multinational Enterprises by the OECD, principles of responsible organizing as initiated by ISO (26,000) or reporting principles as initiated by the Global Reporting Initiative (GRI) (Van Tulder & Van Mil, 2023). The extent to which the SDG agenda can lead to impact on sustainable development thus can become testimony of the question whether a ‘principles-based’ approach can be better, worse, complementary or equal to a ‘rules-based’ approach towards corporate action on sustainable development.

## Slow Progress, and What is Needed?

The results of the first years of the SDG principles-based governance experiment show a mixed picture. Despite widespread support for the goals by almost all leading companies, implementation trails behind expectations and ambitions at the macro-level of analysis (van Tulder & Van Mil, 2023). Since 2015, progress on the SDGs has been slow and, since 2020, even negative in many areas. A 2018 UN global check-up report already warned that progress on the SDGs proved uneven “across regions, between sexes and among people of different ages, wealth and locales, including urban and rural dwellers”, and was not moving fast enough on almost all accounts (UN 2018). Conflict, war and violence were identified as significant exogenous barriers to poverty eradication and sustainability, while progress had not yet reached the people who need it most. But could this situation be attributed to corporate behavior? One particularly relevant finding for assessing the potential role played by companies, was that they were insufficiently succeeding in integrating the SDGs in their core activities and, most importantly, in engaging in meaningful collaboration with other societal actors. The UN Global Compact Progress Report 2019, for instance, found that while 71% of the CEOs recognize the critical role that business could play in contributing to delivery of the SDGs, a mere 21% believed that business is indeed playing that role (UN Global Compact 2019).

Despite these findings, the SDGs can still be considered the most sophisticated principles/goals-based approach available. In a unique confluence of circumstances—which would probably not have been possible one or two years later—the SDGs in 2015 provided a common agenda. This agenda is supported by a coalition of stakeholders willing to coordinate action and collect and harmonize relevant databases around each of the 230 indicators constituting the SDG framework. The latter ambition was organized by the creation of so-called ‘custodian agencies’ like the World Bank, the OECD or specialized organizations like the International Organization for Migration (IOM) or UNESCO. They all promised to work on a unifying scheme of common indicator development of each SDG and the organization of voluntary reporting to keep track of stages of transition.

These efforts have seriously increased the analytical intelligence of the world, by producing a large variety of interim evaluation studies, corporate reports, NGO studies, SDG benchmarks, SDG repositories and the like (cf. Van Tulder & Van Mil, 2023). But they also showed the existence and nature of a sizable gap between intention and realization, which prompts the need for further studies on root-causes and ways to proceed at multiple levels of analysis. Some observers contend that these findings illustrate the failure of the whole SDG exercise (Deacon, 2016; Buhmann, Jonsson and Fisker 2018; c.f. Waage et al., 2015). Others have taken the opposite view that the SDGs are more needed than ever: thus, the plea for a Decade of Action by the UN.

Whatever perspective is taken, however, their holders probably would agree that one of the biggest normative and strategic challenges lies in the middle: how to link micro-level actions with macro-level demands and outcomes through multi-stakeholder and partnering strategies.

## The Case for Collaboration in Principles-Based Approaches

The critical importance of stakeholder engagement and partnerships was already acknowledged in the basic set-up of the SDGs. The United Nations department of Economic and Social Affairs (UNDESA)–- which acts as the Secretariat for the SDGs—highlighted the critical importance of stakeholder engagement and partnerships as follows: “Sustainable development decision making requires broad participation of all. The Division therefore aims to support the effective participation of Major groups (as defined in Agenda 21) and other stakeholders in the UN political process, including through efforts to build their capacity, knowledge and skills base” (UN 2015a).

Agenda 2030, in their terms, has brought clarity about the common purpose that mankind needs to achieve in collaboration to achieve the goals of the agenda and a request for all, including business organizations, to respond to the threats to sustainability. This approach builds on cross-sector partnerships as a key enabler and the principal way forward to serve sustainable development goals and/or address wicked problems and common good challenges (Austin & Seitanidi, 2014; Waddock, Meszoely, Waddell and Dentoni 2015). Wicked problem is a term that reflects the true nature of the SDGs; they are complex issues and interconnected (van Tulder, 2018; Van Tulder & Van Mil, 2023). As highlighted in previous publications, multi-stakeholder initiatives and partnerships are complex and dynamic arrangements which may be subject to specific contextual circumstances (Utting & Zammit, 2009) and additional research has been suggested to study multi-stakeholder arrangements (Eweje, Sajjad, Deba Nath and Kobayashi 2021). While the need for collaboration has been widely acknowledged (Albrectsen, 2017), further research is needed to reveal the uniqueness of each industry, sectors, and partnerships. Several publications have highlighted the challenges in the implementation of SDGs in strategies and operations of business organizations as well as the financial, reputational and organizational consequences thereof (c.f. MacDonald et al., 2018; Rashed & Shah, 2021; Soberón et al., 2020). However, former research has not yet sufficiently answered the question to what extent businesses will need to change and adapt in order to incorporate and achieve the SDGs. The SDGs, formulated as global goals, ask for actions on the micro level (organizations) and the meso level (networks, industries) to achieve results on the macro-level (countries, continents, global sustainability). The demand for actions has an impact on the way how to strategize and organize the approach to these goals. This special issue contributes to multi-stakeholder literature by further studying the role of partnerships and multi-sector alignment, operational perception alignments, and finally, strategic and goals alignment to facilitate change.

One of the biggest bottlenecks, therefore, appears in the practical elaboration of the SDG agenda in concrete partnering strategies—in particular when partnerships are organized across societal sectors (bringing together state, civil society and market actors in varying constellations). Effective cross-sector partnerships (CSPs; c.f. Bryson, et al., 2015; Selsky & Parker, 2005; van Tulder et al., 2016) prove difficult to operationalize and to sustain. A global survey on ‘external engagement’ (McKinsey, 2020), for instance, found that nearly 60 percent of CEOs rank stakeholder engagement among their top three priorities. However, the survey also indicates vast ‘intention-realization’ gaps, with merely 7% of respondents stating that their organization regularly aligns the interests of stakeholders and their business. Moving on from external stakeholder engagement to a committed, formal, and embedded CSP strategy proves even more challenging—which probably explains why many companies still underutilize the potential of partnerships. The UN Global Compact (2020) assessed that only 52% of their signatories are engaging in partnership projects with public or private organizations, whilst endorsing that multi-stakeholder collaboration is key to achieving the transformations and systemic effects needed.

Cross-sector collaboration is complex and ‘collaborative advantage’ challenging to create and harness (Bryson, et al., 2016), which is among the prime reasons why progress on the SDGs has been slow. In 2019, UN DESA concluded that despite the strong rhetoric and overwhelming efforts put into partnering around the world, “the reality is that we are still only scratching the surface in terms of the number, and quality, of partnerships required to deliver the SDGs” (UNDESA, 2019). Undoubtedly, the Agenda 2030 has triggered business research into understanding obligations, contributions and impact of business engagement in sustainability goals. Academic literature on SDGs and company involvement therein unveils (c.f. Montiel et al., 2021; Pizzi et al., 2020) that there is a tendency to focus on certain aspects of company involvement in SDGs and rather apply a macro-perspective or a “global view” (ibid. 13). Other aspects that are prominent in business research are strategic and performance management questions (ibid.). However, so far, the relationship between micro-level initiatives of businesses with meso-level factors and aspects of collaboration, on the one hand and consequences for sustainability on the macro level is under-researched and needs attention to understand the processes that take place in multi-stakeholder engagement.

### Principles-Based Approaches to Complex Challenges: the Need for Stakeholder Engagement

At face-value, the SDG agenda seems to be a normative agenda, but can it also serve as a strategic agenda for companies? An important aspect of business strategies is survival and business success, also in terms of financial and organizational viability. Business sustainability is dissimilar from social sustainability. However, the important question in this context is to show that business organizations should engage in social sustainability goals. That is also posing questions about the business perspective about business roles in society. And taking on social responsibilities may imply adopting an ethical perspective to business and their goals. What perspective does the SDG agenda imply?

As the political orientation of the UN demands—and the choice of words in the preamble underlines—the Agenda 2030 is foremost a normative agenda whose goals are not to be understood as instrumental goals but as absolute goals, i.e. the goals of the Agenda 2030 are directly related to universal human rights. And they go beyond that to describe intact nature and animal welfare as desirable.

The achievement of Agenda 2030 is partly impeded by its overall complexity and all the issues related to it (Waddock, Meszoely, Waddell, Dentoni, 2015). Organizations are limited in the support they can offer to innovate towards accomplishing the SDGs. The intricacy of the 17 goals and the diversity of stakeholders related to issues addressed by organizations can be seen as a positive force as well as a threat. The diversity of stakeholders affected forces organizations to pursue multi stakeholder engagement and collaboration in order to achieve the SDGs (Rotheroe et al., 2003). On the other hand, pre-existing institutional arrangements and procedures for facilitating or fostering collaboration between multi-stakeholders is not available and does not even seem to be possible. In many cases, collaboration for the SDGs can even be typified as a “[…] result of emergent and unforeseen interorganizational dynamics […]” (vv, Whiteman and Parker 2019: 367). The United Nations acknowledges that progress on the SDGs agenda is too slow, and asks how the pace for change can accelerate. The voluntary nature of the SDGs, the need to clarify organizational and individual moral and ethical obligations, the absence of legal enforcement and sanctions, and the lack of formal processes to ensure the accomplishment of the goals (Biermann et al., 2017; Bowen et al., 2017) mean that the SDGs are often perceived as recommendations and their targets are in need of being legally enforced with a common legal agenda (Persson, Weitz and Nilsson 2016; Van Tulder & Keen, 2018).

There are several reasons why the SDGs can be considered strategic and instrumental as well as normative and absolute.

(A) [a] At least for the UN, the goals of the 2030 Agenda are SMART in the sense of Peter Drucker (1954) and thus follow the operational logic of business organizations, which are to a large extent guided by clearly formulated goals (Veggeland, 2014). The goals are *specific* because each goal describes a clear object, they are *measurable* because in some cases the target is concretely specified (e.g., zero poverty, zero hunger), and in other cases a benchmark is available in the form of the status quo. The goals are *achievable*, even if their attainability is not guaranteed; they are *relevant* because they contribute directly to the realization of human rights. Finally, they are *time-bound*, which is already given by the Agenda's deadline of 2030. However, it is questionable if the goals can be translated directly into SMART business goals.
(B) [b] Moreover, the goals remain also vague in some important respects. It is not clearly defined who has to do what, how the measures to achieve the respective goals are to be financed, and who can assess whether the goals have been achieved. In connection with this, no sanctions are named, either positive or negative.
(C) [c] Furthermore, there exist two tensions in setting the goals. First, the goals are about social and environmental sustainability concerns; at the same time, the goals establish cooperation between different sectors and forms of organization. These goals are very clearly different from typical business goals (greater sales, greater market share, increased efficiency, customer satisfaction, or employee satisfaction), but at the same time they are formulated in such a way that companies should commit to them and collaborate in their implementation. Second, these goals are formulated as normative goals, but they are written for actors with a strategic agenda.
(D) [d] Finally, the SDGs explicitly refer to “multi-stakeholder partnerships and voluntary commitments”. This refers to two terms that are discussed very prominently but also controversially in the CSR and business ethics literature. The stakeholder concept was first introduced as a strategic management term by Freeman (1984). Freeman (1999) himself refers to the distinction between a descriptive, normative, and instrumental theory, and the discussion of whether stakeholder theory is normative or instrumental or even strategic is not settled (c.f. Freeman et al., 2020; Reed, 1999). The second concept that is contested in the CSR literature is that of voluntarism (c.f. Gatti et al., 2019a, 2019b; Gössling, 2011). Until 2011, for example, the European Commission held on to the notion of voluntariness in the definition for CSR and then dropped it (c.f. Gatti, Seele and Rademacher 2019).

The notion of stakeholders and the necessity to engage them towards common ethical and responsible actions to help achieve the SDGs has been extensively discussed in literature. Most stakeholders seem to agree on the importance of the 17 SDGs and on their 169 sub-targets. That said, what will determine the effectiveness of any specific intervention will be the way the goals and targets are interrelated and their complexity; the difficulty of addressing what should be done that also requires legal reinforcement; determining what contributes to moral and/or ethical value at the social level (rather than just the profit motive or business operations); and the ability for involved or affected stakeholders to work together towards a common vision will determine the effectiveness of the chosen intervention (Van Tulder, 2018). Due to their intertwined high level of complexity, SDGs are described as wicked problems requiring cross-sector partnerships, inclusion of multi-stakeholder’s perspectives, and involving different partnerships to create systemic change (Van Tulder & Keen, 2018). Working with the SDGs reveals the importance of partnerships to help address wicked problems.

Even though the logics of the two sectors—the UN on the one hand as an international organization representing governments, not-for-profits, and civil society; and business enterprises on the other hand as market-oriented actors—are different, the SDGs aim to provide a translation service or function to increase the willingness of companies to collaborate—including across sectors—on solutions to the most pressing problems facing the world’s population. In doing so, the SDGs offer important assistance in substantiating what sustainability means; at the same time, however, companies are offered room for maneuver, e.g., regarding which of the 17 goals they will contribute to. Moreover, the way in which business enterprises contribute is also not specified in terms of content. The 2030 Agenda offers important recommendations for implementing the idea of cooperation.

But this recommendation is at the same time a major challenge and management task. For it also means that management should understand and appreciate the relationship between partnership and multi-stakeholder engagement and attach significance to it for decision-making. This also formulates a task for management that has little to do with intra-organizational optimization and more to do with cross-level, cross-organizational, and cross-communication skills, and a willingness to commit to sustainability. In addition, there is the task of recognizing the importance of dialog and communication between different groups of stakeholders to ensure transparency and successful results. This includes dealing with the complexity of defining stakeholders, since stakeholders are a socially constructed phenomenon (Fineman & Clarke, 1996; Winn, 2001). Individuals cannot be assumed to belong to only one group; they often belong to more than one group, and stakeholder groups are heterogeneous (Crane & Livesey, 2003; Gao & Zhang, 2001; Winn, 2001). Identifying stakeholder groups and describing their characteristics and what they mean for relationships (Bryson, 2004; Mitchell et al., 1997) is a highly complex managerial task. This poses the interesting challenge of addressing the empirical question of which organizational and strategic management methods business enterprises do or should use to successfully work on the implementation of the SDGs, whereby the question of success also refers to the two references mentioned, namely the success of the respective enterprise on the one hand and the achievement of the societal goals of the Agenda 2030 on the other.

#### **Contributions to This Special Issue**

The call for papers for this special issue was published in 2020. The nine articles presented in this issue contribute to understanding conditions and meaning of MSEs for the SDGs. To document these contributions, we analyzed the research papers in terms of the following points, which are also listed in Table 1

- What challenge to sustainability does the article address?
- What are the main results?
- What data were used and how did the authors approach the research problem?
- What theory and method were used to analyze the data and answer the research question?
- What context-specific information was provided about the research setting?

**Table 1:** Approaches to multi-stakeholder collaboration for the UN SDGs

	(first) Author	Sustainability challenge	Answer RQ	Data/Approach	Method/Theory	Context specificities
1	Simona Fiandrino	Contributions of companies to the UN Agenda 2030	Success factors: Understanding interconnectedness of goals and inclusiveness of actors; focus on core business, stakeholder orientation, deliberative democracy and meta-governance structure	Conceptual paper to develop a theoretical model	social impact theory (SIT)	-
2	Anthony Alexander	SDGs focus and help for dealing with wicked problems	Importance of data and performance evaluation for addressing wicked problems; participatory and technocratic approaches	Case study with interviews across a range of stakeholders	decision theory to systems theory,	Deforestation in Supply Chains
3	Ozgu Karakulak	The influence of partnership scope on coping of MSPs with complexities	A function-oriented scope helps filter the relevant external and internal complexities, whereas the issue-oriented scope magnifies the complexities	Qualitative data on four MSPs	Comparative case study	Global health partnerships
4	Ethiopia Legesse Segaro	Impact of government intervention and MSPs for SDGs	Stakeholder collaboration helps to achieve SDGs	23 in-depth interviews with different stakeholders	text analysis	international entrepreneurship in African frontier markets
5	Amanda Williams	Engagement of private organizations in strategic multi-stakeholder SDG platforms	Conceptual model of three stages: platform formation, innovation and scaling. An ambition is to shift the value framing from profit outputs to SDG impacts and outcomes	Comparative Case Study	exploratory research, data triangulation	Private industry Danish and Swiss Collaboration
6	Samuel Petros Sebhatu	Use of the SDGs for guiding stakeholder engagement for transformative change?	Preliminary framework: navigating the network for value creation; sustainable societal practices can contribute to a broader view of business transformation, and vice versa	Case data on two cases from 2012 and 2020	theory testing and refinement approach	two cases: Löfbergs and IKEA
7	Laura Mariani	The organization of multi-stakeholder collaboration for the SDGs	NPOs and NGOs as pivotal partners representing government positions	qualitative data of four innovation initiatives across Europe	multiple-case study analysis	energy and food consumption
8	Wendy Stubbs	Interaction between purpose ecosystem actors to achieve the SDGs	Changing the purpose of business and integrating the goals into operations and engagements with stakeholders contributes to achieving the UN SDGs	12 organizations in Australia and six in the UK	Exploratory research approach; content analysis	emerging purpose ecosystem
9	Leopoldo Gutierrez	Outcomes of different stakeholder engagement pathways for innovation relevant for the SDGs	Typology of six different stakeholder engagement strategies: Different stakeholder engagement strategies do deliver different outcomes	Datasets on innovation studies	hypothesis testing survey	Spanish Technological Innovation Panel

The contributions selected for this special issue reflect the desire of international researchers to add empirical substance to claims that multi-stakeholder engagement offers relevant approaches to addressing sustainability challenges. The contributions also reveal that multi-stakeholder engagement ‘with a common purpose’ such as the SDGs allows for more focused and directed thinking about complexity.

To navigate the challenges of all the issues involved in achieving Agenda 2030 (Waddock et al., 2015), principles-based collaborative strategies in support of the SDGs are recommended by all authors. Three strategic levels are discussed by the authors: The role of partnerships and multi-sector alignment, operational perception alignments, and finally, goal and strategic alignments to facilitate change.

### The Role of Partnerships and Multi Sector Alignment

The role of partnerships and multi sector alignment is discussed extensively in the submissions as it has the potential to move beyond the fragmented configurations that currently prevail and help increase and align organizational fit between the complexity of the issue and the potential partners. Previous research highlighted the critical importance of ecosystem management (Dietz 2003) and describes the necessity of societal cross-sector collaboration in support of sustainability (Heuer 2011). Similarly, the importance of the ecosystems and their relevance to facilitate multi-stakeholder collaborations is discussed in this issue (Stubbs, Dahlmann and Raven 2022, THIS ISSUE).

All contributions highlight the importance of multi-stakeholders’ relationships. Examples are diverse in terms of geography and industries. An interesting example is the relational coupling of stakeholders in Ethiopia designed to facilitate the achievement of sustainable prosperity that benefits local and international communities in a context of severe poverty and liquidity constraints. It reveals the importance of cooperation-facilitating agencies, dialogues and collaboration across businesses, governments, and NGOs (Legesse Segaro and Haag 2022, THIS ISSUE).

Additionally, to prevent risks associated with power asymmetries among sectors (Waddell, 2000), differing notions of trust (Selsky & Parker, 2005) and turbulent cross-sector relationships (Trist, 1983), the importance of non-profit organizations potentially acting as meta-governors of collaborative innovation for sustainability is highlighted in several contributions (Martini, Rivellato, Martini and Marafioti 2022, THIS ISSUE). The role of the non-profit sector has already been highlighted in past contributions as potentially incubators for sustainability and social movements (Heuer 2011).

### The People Who Facilitate These New Strategies Will Need Specific Cognitive Competencies.

The People Who Facilitate These New Strategies Will Need Specific Cognitive Competencies. “Most of all, we need to understand that humankind and the natural environment are both part of the ecosystem” (Heuer 2011, p. 219)**.** Literature previously studied the main characteristics of an ecosystem management approach: it should be holistic, interdisciplinary, goal-oriented, participatory and designed to help people realize how they are part of the ecosystem, and not separate from it (Slocombe, 1993). To support such participative and collaborative ecosystems for Agenda 2030, the choice of the facilitators’ profile is therefore highly relevant. In addition, the type of communication used by these agents of change is essential to engage and facilitate any collaboration. To be successful, multi-stakeholder engagement must be participatory and requires a thorough understanding of processes of inter-organizational decision-making integrating emotions and the role of ethical values (Alexander et al. 2022, THIS ISSUE).

Research shows how specific individuals’ profiles can help overcome the difficulties of implementing of strategic responses to Agenda 2030 challenges. When faced with changes, cognitive requirements are high to create coalitions and engage across industries, across the supply chain, and sectors. Specific agents’ profiles can help create interconnectedness and inclusiveness (Fiandrino, Scarpa and Torelli 2022, THIS ISSUE). The cognitive roles played by key actors who can take multiple roles and engage with diverse stakeholders is critical. Some of these roles are described as sponsors, who have the needed influence to get organizations involved; others are champions, who are there on a day-to-day basis to make sure good things happen (see also Bryson et al., 2015). Other are influencers and facilitators, who see themselves as ‘enablers’, ‘pioneers’ and ‘critical friends’ (Stubbs et al. 2022, THIS ISSUE). Those playing these roles will jointly with others help engage dialogue, sharing, and the more toward more shared leadership (see also Quick 2015). They will help navigate the complexities by helping gain access to information, knowledge, resources and skills. Together they will help maintain a focused agenda and deliver the needed results.

When engaging with several actors who sometimes have no experience of working together and do not share the same organizational priorities (Gray & Purdy, 2018; Innes & Booher, 2010), a common and collaborative approach can be very hard to create. To help cope with the complexity of the process, communication is key and actors will benefit from clear messages versus mixed messages (Karakulak, Stadtler 2022, THIS ISSUE).

## Engaging in the alignment of Operations and Perceptions Requires A Clarified Strategic Plan

Ecosystems are complex, dynamic and subject to an immense number of internal and external relationships (Heuer 2011). Creating coalitions where diverse stakeholders will share resources and engage together is extremely uncertain and presents unique challenges. Clarifying their strategic plans and helping each organization develop a sense of strategic belonging by engaging them on a common strategic agenda and targets is a way to avoid fragmentation and improve interagency collaboration­­-a common vision to adhere to, one that will help them pool their resources and work on a similar journey. To this end, Agenda 2030 and the 17 SDGs, along with their 169 sub-goals provide an essential framework to help define a common global agenda for nations, organizations and civil societies (Williams and Blasberg 2022, THIS ISSUE). The UN SDGs help frame the tools, discussions, and interactions in a global discourse on humanity’s challenges (Stubbs et al. 2022, THIS ISSUE).

To ensure the success of the new strategy designed around the Agenda 2030, many implementation challenges must be addressed. To facilitate stakeholder engagement, (Gutierrez, Montiel, Surroca and Tribo 2022, THIS ISSUE) have developed a typology of six different strategies for engaging with stakeholder groups: opportunity exploration, uncommitted diversification, rainbow war, rainbow washing and progressive learning. When facing difficult issues, it is of utmost importance to clarify the common purpose and benefits to be gained from collaboration (Huxham and Vanagen 2005; Bryson et al., 2016) in order to facilitate the steering and navigation that is needed in the new landscape of SDG’s challenges (Sebhatu and Enquist 2022, THIS ISSUE).

## Conclusion and Areas for Further Research

The call for proposals not only triggered a large number of potential contributions, but also showed that there are still major areas of research to be covered in the coming years. The aim of a special issue is not only to showcase present research, but also to stimulate future research. What can we learn from the present ‘harvest’ of special issue papers? Most of the work that is published in this special issue further builds on findings and discussions known from the multi-stakeholder and partnership alliance literature. The impact of multi-stakeholder processes is more difficult to research and thus is not yet well covered. The present research mostly focuses on interaction between partners, but not really on the ethical principles – like procedural justice (cf., Page et al., 2015)—or governance principles—like hybrid governance or ‘governing through goals’—that might provide answers to the ultimate impact that effective partnering can have on a number of focused goals (cf. Van Tulder et al., 2016).

Extant research on multi-stakeholder processes for the SDGs seems to favor governance over ethics and pragmatics over principles, and reactive (negative duty) approaches over proactive (positive duty) approaches. This tends to underestimate the principles-based potential of the SDGs agenda: common goals and principles that require a more pragmatic angle towards reaching goals. A future research agenda in support of the ‘Decade of Action’ (which can improve the contribution of business research to the much-needed acceleration of the SDGs) then can be as much strategic as ethical and normative, while the engagement of multi-stakeholders can be as much practical as principled.

**Fig. 1:**
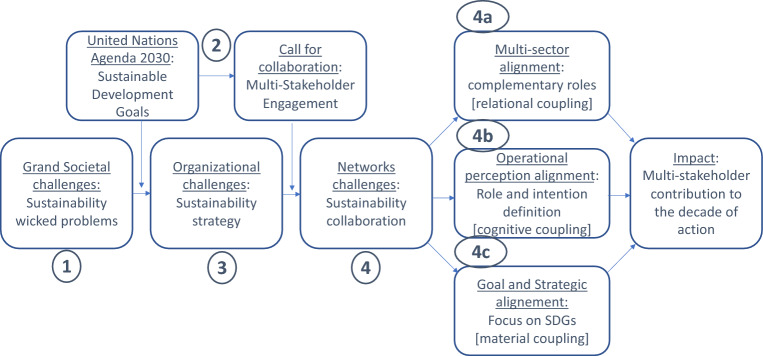
Navigating principles-based collaborative strategies in support of the SDGs

Navigating research around relevant themes can then be guided by the following analytical scheme that summarizes the state-of-research in business ethics research on SDGs as shown in Fig. 1.

Furthering the SDG agenda and contributing to the Decade of Action, presents a number of challenges to researchers:

1. Neither the necessity for cross-sector collaboration to achieve the SDGs nor the importance of private sector contributions presents a particularly major issue for further ethical research. The relevance of collaboration for the creation of common goods is undisputed.
2. The need for private engagement and collaborative (multi-sector) efforts in dealing with complex/wicked problems in a ‘fair’ and ‘equitable’ manner is also widely acknowledged. So-called ‘second generation’ wicked problems and complexity theory (Head 2015; Termeer et al., 2019) shows that wicked problems cannot be solved per se, but can be addressed by multi-stakeholder arrangements and by the involvement of private actors.
3. Effective MSPs for the SDGs partly depend on the translation of sustainability strategies into effective network and collaborative strategies aimed at achieving longer term impact on complex issues such as the SDGs and effectively contribute to the Decade of Action. The translation of sustainability strategies into network and collaborative strategies poses a number of challenges; for example, how to use the nexus potential of the SDG agenda (cf. Stockholm Resilience Centre) while translating this to individual corporate action (cf. Van Zanten & Van Tulder, 2020); how to create the proper coalitions ex-ante and design a proper theory of change and ‘developmental evaluation’ principles that also leaves room to learn from the experience of the partnership to improve its impact along the way (Patton, 2021); and how to overcome the gap between strategic intent (and normative absolute principles) and operational realization (and operational instrumental business principles).
4. These concerns boil down to the translation of corporate strategies into effective partnering strategies that can reap ‘collaborative advantage’ for common goals such as the SDGs. Here we see the biggest gaps in our understanding of relevant management practices in support of the SDGs. For further research we propose to focus on three types of alignment- and related coupling—questions which also concern ways in which business ethics research on the principles-based initiatives such as SDGs can profit from interdisciplinary crossovers from a variety of scientific disciplines, in particular (1) strategic management (Gond et al., 2018), (2) human resources management, (3) political economy and governance studies, and (4) organization sciences. The three couplings may be described as follows:

*Multi-sector alignment and relational coupling*: linking the relationships among partners to highlight, embrace and commit to the SDGs addressed;

*Operational perception alignment and cognitive couplin*g: linking implementation challenges related to translating intentions into realizations and the concrete cognitive requirements of effective managers. 

*Goal and strategic alignment and material couplin*g: linking present materiality questions of strategic action—such as strategic plans, KPIs, and business models—to fully integrate addressing the SDGs into corporate strategies.

*[ad.4a] Multi-sector alignment and relational coupling *The cross-sector partnering literature already shows great potential in addressing ways to look at the alignment between complex issues and corporate strategies. The extent to which partnerships can create sufficient ‘complementary’ value by aligning ‘coalitions of the needed’—instead of the relatively fragmented ‘coalitions of the willing’ that presently prevail—can increase the organizational ‘fit’ between the complexity of the issue and the partnering configuration. This presents a promising area of further research (cf. Austin & Seitanidi, 2014; Van Tulder & Keen, 2018). More interdisciplinary work is required. In particular, business management literature and scholarship would also benefit from paying more attention to the public and nonprofit management and political science literatures, where collaboration, governance, and social movements have been important research topics for decades. To the extent that governments, government agencies, and nonprofits are necessary for achievement of the SDGs, the collaborative advantage of building of these literatures should be pursued. Given the urgency of the challenges, time should not be wasted on reinventing the wheel.

*[ad. 4b] Operational cognitive coupling: *The strategic management literature talks about strategic ‘tinkering’ (Mintzberg, 1987) as a relevant frame to assess more or less ‘salient’ implementation strategies. The gap between ‘intention’ and ‘realization’ is not necessarily a ‘moral gap.’ It may simply be a part of day-to-day practice, especially in the case of organizations trying to address complex issues in collaborative efforts. Thus, despite good intentions, unintended outcomes are likely to emerge out of these innovative processes designed to address complex situations. An adequate cognitive (collaborative) mindset is needed to manage these processes. Clarification of the process, the animating vision, and dialogue to discuss hurdles and strategies are key factors supporting intended outcomes (Legesse Segaro, E. et al., THIS ISSUE 2022).

*[ad.4c] Strategic and material coupling: *Addressing the SDGs effectively also requires the adaptation of more traditional strategic management approaches to situations that go beyond what any organization can accomplish by itself, and where no organization is wholly in charge. Strategic management of a single organization involves a fairly well-known set of tasks and often involves the development of a strategic management system to ensure direction, alignment, and commitment across the organization (Drath et al., 2008; Whittington & Yakis-Douglas, 2020). Strategic management of collaborations and even social movements, though less studied, is becoming more common and necessary, given the boundary-crossing challenges facing the world. Leading several organizations to achieve a common purpose has been called leading strategy management-at-scale, meaning the scale of the challenge to be addressed (Bryson et al., 2021). Such cross-boundary issues include the global COVID-19 pandemic and how to achieve the SDGs. Such issues occur within a shared-power, no-one-wholly-in-charge environment and demand a response from multiple organizations. Various strands of reasonably aligned, if not directly coordinated, effort are required. Two complementary approaches to strategy management-at-scale include collaboration itself, and beyond that, community organizing, coalition building, and advocacy.

One popular approach to collaboration in the US is called Collective Impact (CI), which became quite popular after a now widely cited article by John Kania and Mark Kramer with a catchy title, “Collective Impact,” in a 2011 issue of the *Stanford Social Innovation Review*. The authors asserted that achieving CI requires a disciplined cross-organizational and cross-sector approach on a scale that matches the challenge. They argued that “five conditions” were necessary to achieve collective impact (39–40): a common agenda, shared measurement system, mutually reinforcing activities, frequent and structured communications, and a “backbone organization.” The approach has been modified since, but the basic idea still has merit (Bryson et al., 2021; c.f. Kania et al., 2022).

The most serious critique of the CI approach is that it has great difficulty achieving deep-seated system change, equity, and justice (e.g., Christens and Inzio 2015; Wolff, 2016). This critique draws limits around the situations in which CI is likely to be helpful. Specifically, really addressing issues of equity, social justice, and system change requires community organizing, coalition building, and advocacy (Wolff et al. 2016; Almeida, 2019). CI initiatives and community organizing efforts of course can be mutually reinforcing. System changes that require better alignment and inter-organizational service coordination may be achieved relatively quickly using a CI approach. When “changes require concessions from entrenched interests, or reorganization and reorientation of existing institutions,” community organizing, coalition building, and advocacy are “likely the more effective approach” (Christens & Inzeo, 2015, 431.) When both kinds of changes are needed, the two approaches can be complementary.

The nine contributions that were selected for this special issue help address the question of how collaboration and communication in multi-stakeholder contexts can contribute to effectively addressing global sustainability challenges as defined by the SDGs. The articles show that while multi-stakeholder approaches can produce significant gains, the approaches are never particularly easy to pursue. We have outlined three major research pathways that address the role of members to a partnership, including alignment across sectors, operational perception alignment, and goal and strategic alignment. In addition, our model discusses the connections between the grand challenges behind the Agenda 2030 on the one hand, and contributions of businesses to these goals, on the other. Finally, all contributions THIS ISSUE contribute to the unfolding of a research agenda that centers around the question about the possible impact of multi-partner collaboration on sustainable goals.

## Declarations

### Research Involve Human and Animals

The research does not involve human and/or animals.
